# Characteristics and Distribution of Intracranial Aneurysms in Patients with Autosomal Dominant Polycystic Kidney Disease Compared with the General Population: A Meta-Analysis

**DOI:** 10.34067/KID.0000000000000092

**Published:** 2023-02-20

**Authors:** Julien Haemmerli, Sandrine Morel, Marc Georges, Fadi Haidar, Fouad T. Chebib, Akio Morita, Kazuhiko Nozaki, Teiji Tominaga, Anatoliy V. Bervitskiy, Jamil Rzaev, Karl Schaller, Philippe Bijlenga

**Affiliations:** 1 Division of Neurosurgery, Department of Clinical Neurosciences, Geneva University Hospitals and Faculty of Medicine, University of Geneva, Geneva, Switzerland; 2 Department of Pathology and Immunology, Faculty of Medicine, University of Geneva, Geneva, Switzerland; 3 Division of Nephrology, Geneva University Hospitals and Faculty of Medicine, Geneva, Switzerland; 4 Division of Transplantation, Geneva University Hospitals and Faculty of Medicine, Geneva, Switzerland; 5 Division of Nephrology and Hypertension, Mayo Clinic, Rochester, Minnesota; 6 Department of Neurological Surgery, Nippon Medical School, Tokyo, Japan; 7 Department of Neurosurgery, Shiga University of Medical Science, Otsu, Japan; 8 Department of Neurosurgery, Tohoku University Graduate School of Medicine, Sendai, Japan; 9 The “Federal Centre of Neurosurgery” of the Ministry of Health of the Russian Federation Novosibirsk, Novosibirsk Region, Novosibirsk, Russia

**Keywords:** genetics, ADPKD, hypertension, neurovascular, intracranial aneurysms, autosomal polycystic kidney disease, aneurysmal locations

## Abstract

**Key Points:**

- IAs location distribution in patients with ADPKD differ from the ones in non-ADPKD patients
- IAs in patients with ADPKD are more commonly located in the anterior circulation and in large caliber arteries
- Because of IA multiplicity and singular IA distribution, patients with ADPKD represent a special population who need to be closely followed

**Background:**

Autosomal dominant polycystic kidney disease (ADPKD) is the most common genetic condition associated with intracranial aneurysms (IAs). The associated pathophysiology remains unknown, but an association with wall shear stress is suspected. Cerebral arterial location is the principal factor influencing IA natural history. This study aims to compare IA location-specific distribution between ADPKD and non-ADPKD patients.

**Methods:**

The ADPKD group comprised data from a systematic review of the literature (2016–2020, *N*=7) and three cohorts: integrated biomedical informatics for the management of cerebral aneurysms, Novosibirsk, and Unruptured Cerebral Aneurysms Study. The non-ADPKD group was formed from the integrated biomedical informatics for the management of cerebral aneurysms, Unruptured Cerebral Aneurysms Study, International Stroke Genetics Consortium, and the Finnish cohort from the literature. Patients and IAs characteristics were compared between ADPKD and non-ADPKD groups, and a meta-analysis for IA locations was performed.

**Results:**

A total of 1184 IAs from patients with ADPKD were compared with 21,040 IAs from non-ADPKD patients. In total, 78.6% of patients with ADPKD had hypertension versus 39.2% of non-ADPKD patients. A total of 32.4% of patients with ADPKD were smokers versus 31.5% of non-ADPKD patients. In total, 30.1% of patients with ADPKD had a positive family history for IA versus 15.8% of the non-ADPKD patients. Patients with ADPKD showed a higher rate of IA multiplicity (33.2% versus 23.1%). IAs from patients with ADPKD showed a significant predominance across the internal carotid and middle cerebral arteries. Posterior communicating IAs were more frequently found in the non-ADPKD group. The meta-analysis confirmed a predominance of IAs in the patients with ADPKD across large caliber arteries (odds ratio [95% confidence interval]: internal carotid artery: 1.90 [1.10 to 3.29]; middle cerebral artery: 1.18 [1.02–1.36]). Small diameter arteries, such as the posterior communicating, were observed more in non-ADPKD patients (0.21 [0.11–0.88]).

**Conclusion:**

This analysis shows that IAs diagnosed in patients with ADPKD are more often localized in large caliber arteries from the anterior circulation in comparison with IAs in non-ADPKD patients. It shows that primary cilia driven wall shear stress vessel remodeling to be more critical in cerebral anterior circulation large caliber arteries.

## Graphical abstract

**Figure:**
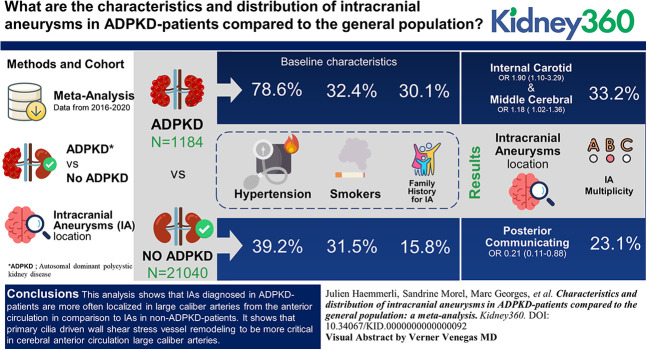

## Introduction

Autosomal dominant polycystic kidney disease (ADPKD) is characterized by several extrarenal manifestations, such as intracranial aneurysms (IAs).^1–10^ The incidence of IAs in patients with ADPKD is higher than the one reported in the general population (4%–40% versus 3%–5%, respectively).^2–4,7,11^ Patients with ADPKD carry a mutation in polycystin-1 or polycystin-2 genes coding for proteins found in the cerebrovascular endothelial layer and in the lamina muscularis.^2,12,13^ One hypothesis regarding the development of IAs in patients with ADPKD is that mutated polycystins affect the expression or the function of the primary cilia present in vascular cells leading to an abnormal vascular fragility.^14^ In arterial endothelial cells, primary cilia are sensors of wall shear stress (WSS), an hemodynamic factor well known to be part of IA formation.^15–17^

Risks factors for IAs rupture have been extensively analyzed in patients not suffering of non-ADPKD.^3,18–20^ Observations from these studies highlight that IA location is the strongest factor associated with IA formation and rupture.^18–22^ More recently, location-based hemodynamic and computed fluid dynamic models have been conducted to understand the appearance and evolution of IA and have demonstrated different patterns of WSS in the cerebral arterial tree.^23–26^ Considering the importance of WSS sensing in aneurysmal disease, we hypothesized that IAs distribution and the diameter of affected arteries could differ between ADPKD and non-ADPKD patients. To investigate differences between demographic data of ADPKD and non-ADPKD patients and differences between characteristics of their IAs, we analyzed combined data from six studies from the literature and three independent cohorts.

## Materials and Methods

### Review of the Literature and PRISMA Statements

**Figure 1:**
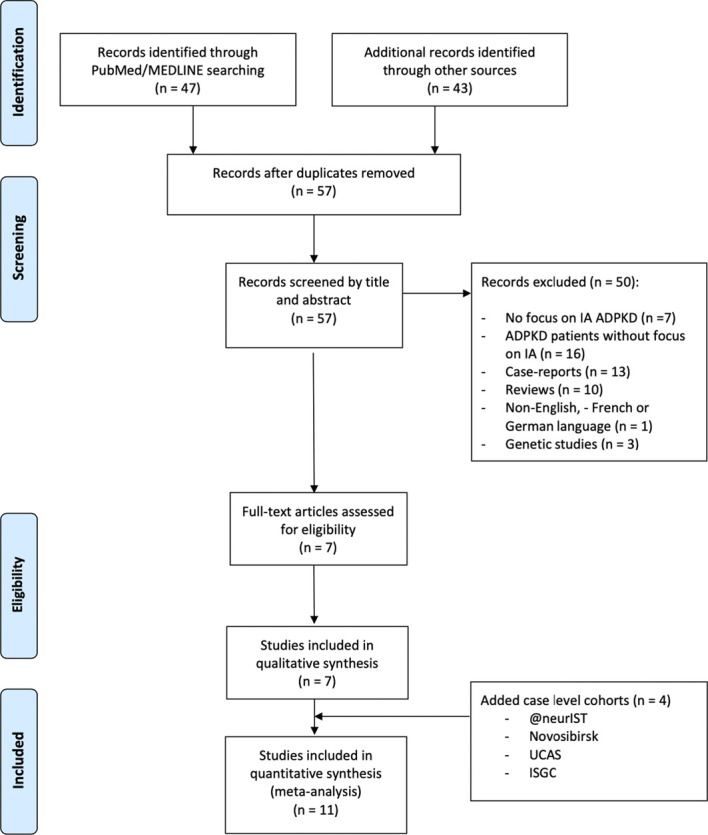
**PRISMA flow diagram.** @neurIST, integrated biomedical informatics for the management of cerebral aneurysms; ADPKD, autosomal polycystic kidney disease; IA, intracranial aneurysms; ISGC, International Stroke Genetics Consortium; UCAS, Unruptured Cerebral Aneurysms Study.

A systematic review of the literature was conducted in three databases (PubMed/MEDLINE and Embase). Figure 1 presents the Preferred Reporting Items for Systematic Reviews and Meta-Analyses (PRISMA) flow diagram used for the purpose of this study.^27^ The literature was searched using the following sequence on PubMed/MEDLINE “(((intracranial aneurysms [Title/Abstract]) OR cerebral aneurysms)) AND ((autosomal polycystic kidney disease [Title/Abstract]) OR polycystic kidney disease [Title/Abstract]).” Search on Embase was performed using the sequence “('intracranial aneurysms':ab,ti OR 'cerebral aneurysms':ab,ti) AND [2016-2020]/py”. Inclusion criteria were (1) series or systematic reviews reporting patients with ADPKD with IA from November 2016 to February 2020, (2) studies on IA treatment of patients with ADPKD, (3) comparison of non-ADPKD versus ADPKD patients with IAs, and (4) articles in English/French/German. Exclusion criteria were (1) case reports or collected data on <3 patients, (2) review, (3) studies reporting patients with ADPKD without detailed information on IAs, (4) studies on IAs without detailed information on patients with ADPKD, and (5) genetic studies. For case-control studies of patients with ADPKD, only patients with ADPKD and IA data were included. No patients with other cystic genetic condition associated with intracranial aneurism were included within the ADPKD group.

### Prospective Data

Data on ADPKD and non-ADPKD patients were collected from three cohorts of patients diagnosed with at least one IA and with a known ADPKD status. Patients with other cystic genetic condition were excluded from all prospective cohorts.

The integrated biomedical informatics for the management of cerebral aneurysms (@neurIST) cohort of the Geneva University Hospitals is a prospective and consecutive collection of clinical, radiologic, and pathologic information on patients with IAs.^12^ Inclusion criteria were (1) newly diagnosed IA between 2006 and 2017 on the basis of angiographic imaging (digital subtraction angiography, magnetic resonance angiogram, or computed tomography with angiogram sequence); (2) patients older than 18 years; (3) presence or absence of ADPKD: the diagnosis of ADPKD was previously made based on enlarged kidney size, the presence of bilateral renal cysts and/or familial history, and made by a nephrologist; and (4) acceptance and signature of consent forms. This study was approved by the Ethical Committee of the Geneva University Hospitals, Geneva, Switzerland, as part of the @neurIST study (PB_2018-0073, previously NAC 07-056). All patients gave their consent and approved for the use of clinical and radiologic data, as well as biologic samples in the field of cerebrovascular research.

The Novosibirsk cohort is a prospective collection of consecutive data only on patients with ADPKD. The applied inclusion criteria were the same as for the @neurIST cohort. This study was approved by the local ethical committee (N°11, researchers Anatoliy V. Bervitskiy and Jamil Rzaev).

The Unruptured Cerebral Aneurysms Study (UCAS) cohort is a prospective collection of data already used in 2012 to study the natural history of aneurysms in the Japanese population. The ADPKD status of patients was recorded and not yet analyzed nor reported.^19^ In accordance with the authors and the local ethical committee, we included the patients with ADPKD from this cohort to the ADPKD group.

The systematic review lacks information on non-ADPKD cases to allow an ADPKD versus non-ADPKD comparison. Only the study by Nurmonen *et al.*^5^ compared ADPKD and non-ADPKD patients. To allow comparing ADPKD patient with non-ADPKD patient characteristics, data collected globally in the context of the International Stroke Genetics Consortium (ISGC) IA working group (International Stroke Genetics Consortium Intracranial Aneurysm Group) was used.^22^ To avoid duplicates, Geneva @neurIST cases were removed from the ISGC dataset for this analysis. All patients from the ISGC, Nurmonen *et al.* study, @neurIST, and UCAS cohorts who were not affected by ADPKD formed the non-ADPKD group. The non-ADPKD group was used as reference group for all comparison when data on non-ADPKD were missing (Table 1).

**Table 1:** Distribution of nonautosomal polycystic kidney disease intracranial aneurysmss per locations from the Finnish, integrated biomedical informatics for the management of cerebral aneurysms, Unruptured Cerebral Aneurysms Study, and International Stroke Genetics Consortium cohorts

Study	ICA *N* (%)	Acom *N* (%)	MCA *N* (%)	Perical. *N* (%)	Pcom *N* (%)	PCir *N* (%)	Other *N* (%)	Total *N*
Nurmonen *et al.*^5^	446 (7)	1332 (21)	2612 (40)	320 (5)	904 (14)	569 (9)	276 (4)	6459
@neurIST	185 (18)	211 (20)	359 (35)	41 (4)	87 (8)	74 (7)	74 (7)	1031
UCAS^19^	1244 (19)	1034 (15)	2415 (36)	267 (4)	1034 (15)	565 (8)	116 (2)	6675
ISGC^22^	1045 (15)	1926 (28)	1709 (25)	238 (3)	1084 (16)	794 (12)	79 (1)	6875
All non-ADPKD	2920 (14)	4503 (21)	7095 (34)	866 (4)	3109 (15)	2002 (10)	545 (3)	21,040

ICA, internal carotid artery; Acom, anterior communicating artery; MCA, middle cerebral; Peri, pericallosal artery; Pcom, posterior communicating; PCir, posterior circulation; @neurIST, integrated biomedical informatics for the management of cerebral aneurysms; UCAS, Unruptured Cerebral Aneurysms Study; ISGC, International Stroke Genetics Consortium; ADPKD, autosomal dominant polycystic kidney disease.

### Studied Parameters

The following demographic parameters were searched for both ADPKD and non-ADPKD groups: (1) age, (2) sex, (3) positive family history of IA, (4) high blood pressure (hypertension), (5) smoking status, and (6) multiplicity of IA.

The following radiologic parameters were searched for both groups: (1) number of IA, (2) IA maximal diameter, and (3) IA location. On the basis of the current literature,^19,21,28^ locations were defined as internal carotid artery (ICA, including ophthalmic and bifurcation segments), anterior communicating artery (Acom), middle cerebral artery (MCA, including M1 segment, bifurcation, and M2 segment), posterior communicating artery (Pcom), pericallosal artery (Peri), posterior circulation (PCir, including posterior inferior cerebellar artery, basilar, and it branches, posterior cerebral artery), and other locations (other).

Aneurysm rupture status was not evaluated for the purpose of this study. In addition, concerning the ADPKD group, neither the renal function at time of IA diagnosis nor the renal transplant status were not recorded as these data were missing in the included literature.

### Outcomes

Basic characteristics of patients with ADPKD with IAs were compared with non-ADPKD patients with IAs. The primary aim was to compare and potentially identify differences in distribution across locations of IAs between the ADPKD and the non-ADPKD cohorts. Secondary outcomes were the demographic data analysis and IA characteristics between the ADPKD and the non-ADPKD populations.

### Statistics and Analysis

**Figure 2:**
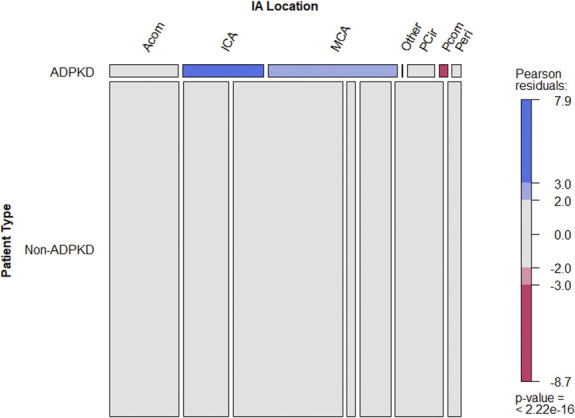
**Mosaic plot depicting the number of IAs in the ADPKD and non-ADPKD groups for each IA location.** Exact numbers and relative proportions regarding IAs by locations groups between Autosomal dominant polycystic kidney disease (ADPDK) and non-ADPKD patients are presented in Table 2. Acom, anterior communicating artery; ADPKD, autosomal dominant polycystic kidney disease; IA, intracranial aneurysms; ICA, internal carotid artery; MCA, middle cerebral artery; PCir, posterior circulation; Pcom, posterior communicating artery; Peri, pericallosal artery.

RStudio was used for the purpose of the analysis (RStudio, version 1.2.5019 running R version 3.6.1). Binary variables were expressed in proportions and analyzed using a chi-squared test and a Fisher exact test. Ordinal variables were expressed in median and quartiles and were compared using the Fisher exact test or Mann-Whitney *U* test when appropriate. Continuous variables were reported for means and SD and were compared using two-tailed nonpaired *t* test. The assessments of differences in distribution among groups were performed using a Kruskal-Wallis test. Hypothesis testing was considered significant for *P* value <0.05. Distribution of IA according to locations was expressed using Pearson residuals, with value ≥2 considered as significant (*P*<0.001). The meta-analysis included all available data. Distribution of IA among locations in the ADPKD group was homogenous between all data sources. Distribution of IA among location in the non-ADPKD group was heterogeneous. It was decided to perform in addition to the pooled data analysis presented in Figure 2 and Table 2, a meta-analysis comparing the distribution of IAs among locations in each study where the data were available and use the data of the non-ADPKD group if non-ADPKD data were missing in this study. For the purpose of the meta-analysis according to locations, odds ratios (ORs) and relative risks were calculated per locations comparing ADPKD-IAs and non–ADPKD-IAs. A fixed effect model was retained when the calculated heterogeneity I^2^ was ≥70%, and a random effect model was accepted for a I^2^ >70%.^29,30^ An OR above 1.0 represents an overrepresentation in the ADPKD group.

**Table 2:** Patients and IAs characteristics in the ADPKD and non-ADPKD groups

COLUMN HEADING	ADPKD	Non-ADPKD	*P* value
**Patients characteristics**			
Total of patients	894	17,864	
Age, yr (mean±SD)	51.32±4.0	56.9±4.9	
Female	608 (68%)	11,575 (65%)	
Tobacco	84/259 (32.4%)	5627 (31.5%)	0.74
Hypertension	703 (78.6%)	6946 (38.9%)	<0.001
IA multiplicity	81/244 (33.2%)	4131 (23.1%)	<0.001
IA-positive family history	78/259 (30.1%)	2831 (15.8%)	<0.001
**IAs characteristics**			
Total of aneurysms	1184	21,040	
Size, mm (mean±SD)	5.1±0.99	6.2±0.62	
Total of IA with location	795 (67.1%)	21,040 (100%)	
Location			
MCA	317 (39.9%)	7095 (33.7%)	<0.001
ICA	198 (24.9%)	2920 (13.9%)	<0.001
Acom	168 (21.1%)	4503 (21.4%)	0.89
Pcom	21 (2.6%)	3109 (14.8%)	<0.001
Peri	22 (2.8%)	866 (4.1%)	0.06
PCir	66 (8.3%)	2002 (9.5%)	0.27
Other	3 (0.4%)	545 (2.62%)	<0.001

IA, intracranial aneurysm; ADPKD, autosomal dominant polycystic kidney disease; MCA, middle cerebral artery; ICA, internal carotid artery; Acom, anterior communicating artery; Pcom, posterior communicating artery; Peri, pericallosal artery; PCir, posterior circulation.

## Results

### Literature Review and Additional Cohorts

The search strategy is presented in Figure 1. A total of 90 studies published between November 2016 and February 2020 reporting patients with ADPKD with IAs were identified. After removal of duplicates and according to inclusion and exclusion criteria, seven studies were included (Table 3). In addition, patients with ADPKD from the @neurIST (21 patients with ADPKD with 45 IAs), Novosibirsk (5 patients with ADPKD with nine IAs), and UCAS (18 patients with ADPKD with 22 IAs) cohorts were included (Table 3). Nurmonen *et al.* conducted a prospective analysis including Finnish patients from a local databank. The authors compared demographic and radiographic data on IAs among ADPKD and non-ADPKD patients from Finland.^5^

**Table 3:** Characteristics of the included studies

Study	Article Type	Origin	Total of Patients	Total of Non-ADPKD Patients	Total of ADPKD-Patients	Total of IAs	Total of Non–ADPKD-IAs	Total of ADPKD-IAs
**Included studies from systematic review of the literature (2016–2020)**								
Nurmonen *et al.*^5^	Cohort	Finland	4436	4383	53	6554	6459	95
Cagnazzo *et al.*^4^	Systematic review	International	563	0	563	679	0	679
Sorenson *et al.*^6^	Case-control	USA	42	0	42	61	0	61
Sanchis *et al.*^11^	Cohort	USA	75	0	75	94	0	94
Kim *et al.*^7^	Cohort	Korea	23	0	23	43	0	43
Wilkinson *et al.*^31^	Cohort	USA	45	0	45	71	0	71
Yoshida *et al.*^10^	Cohort	Japan	49	0	49	65	0	65
**Additional included cohorts**								
@neurIST	Cohort	Switzerland	925	904	21	1076	1031	45
Novosibirsk	Cohort	Russia	5	0	5	9	0	9
UCAS^19^	Cohort	Japan	5720	5702	18	6697	6675	22
ISGC^22^	Cohort	International			0	6875	6875	0
Total			11,883	10,989	894	15,349	14,165	1184

ADPKD, autosomal polycystic kidney disease; IA, intracranial aneurysms; @neurIST, integrated biomedical informatics for the management of cerebral aneurysms; UCAS, Unruptured Cerebral Aneurysms Study; ISGC, International Stroke Genetics Consortium.

Non-ADPKD patients from the @neurIST, UCAS, ISGC cohorts, and the Finnish cohort were compared for IA distribution. Differences were found between the cohorts that are averaged out by pooling the data in a non-APDKD group used as a surrogate to impute for missing values. Consequently, a total of 10,989 patients with 14,165 IAs were included in the non-ADPKD group (Table 3).

### Patients and IAs Characteristics in the ADPKD and Non-ADPKD Groups

A total of 894 patients with ADPKD with 1184 IAs were compared with 17,864 non-ADPKD patients with 21,040 IAs. Table 2 presents the demographic comparison between the non-ADPKD and the ADPKD groups. No difference was found regarding sex and age at diagnosis. A total of 32.4% of patients in the ADPKD group were smokers versus 31.5% in the non-ADPKD group (*P* = 0.74). At the time of IA diagnosis, uncontrolled or poorly controlled arterial hypertension was more frequently observed in the ADPKD group (78.6%) than in the non-ADPKD group (38.9%, *P* < 0.001). In total, 30.1% of ADPKD patients had a positive family history for IA against 15.8% in the non-ADPKD group (*P* < 0.001). Furthermore, multiple IAs were more frequently found in patients with ADPKD (33.2%) than in the non-ADPKD patients (23.1%, *P* < 0.001).

Concerning IAs characteristics between ADPKD and non-ADPKD patients (Table 2), no difference was found regarding IA size at diagnosis between the two groups (5.1±0.99 mm versus 6.2±0.62 mm, respectively). All IA locations were reported for the non-ADPKD group; 795 IA locations were described in the ADPKD group (67.1%). IAs distribution by location in the non-ADPKD group was as follows: MCA 33.7%, ICA 13.9%, Acom 21.4%, Pcom 14.8%, PCir 9.5%, Peri 4.1%, and other 2.6%. In the ADPKD group, in comparison with the non-ADPKD patients, IAs were more frequently found at ICA (24.9%, *P* < 0.001) and MCA (39.9%, *P* < 0.001) and less frequently at Pcom (2.6%, *P* < 0.001). No significant difference was found regarding the Acom location (21.1%, *P* = 0.89) and the Peri location (2.8%, *P* = 0.06). Interestingly, no significant difference was found concerning the PCir location (8.3%, *P* = 0.27). Figure 2 presents the mosaic plot of the distribution of IAs per location using the Pearson residual to assess the strength of the differences observed.

### ORs of IAs Distribution by Location: Meta-Analysis

**Figure 3:**
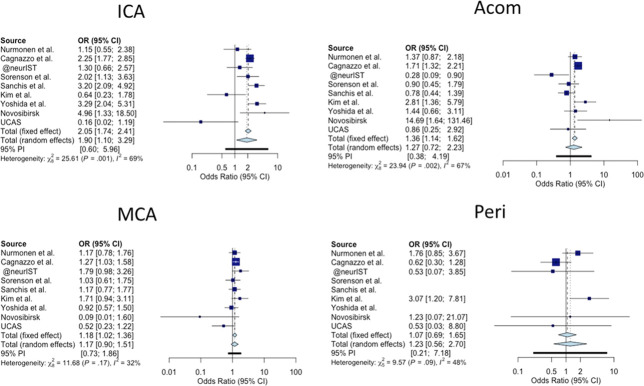
**Forest plot of the IA location distribution at the anterior circulation level.** A fixed effect model was chosen for an interstudies heterogeneity I^2^≤70% and a random effect model for an I^2^>70%. An odds ratio >1 favors the location to Autosomal dominant polycystic kidney disease (ADPDK) condition. Acom, anterior communicating artery; CI, confidence interval; IA, intracranial aneurysms; ICA, internal carotid artery; MCA, middle cerebral artery; OR, odds ratio; Peri, pericallosal artery; UCAS, Unruptured Cerebral Aneurysms Study.

**Figure 4:**
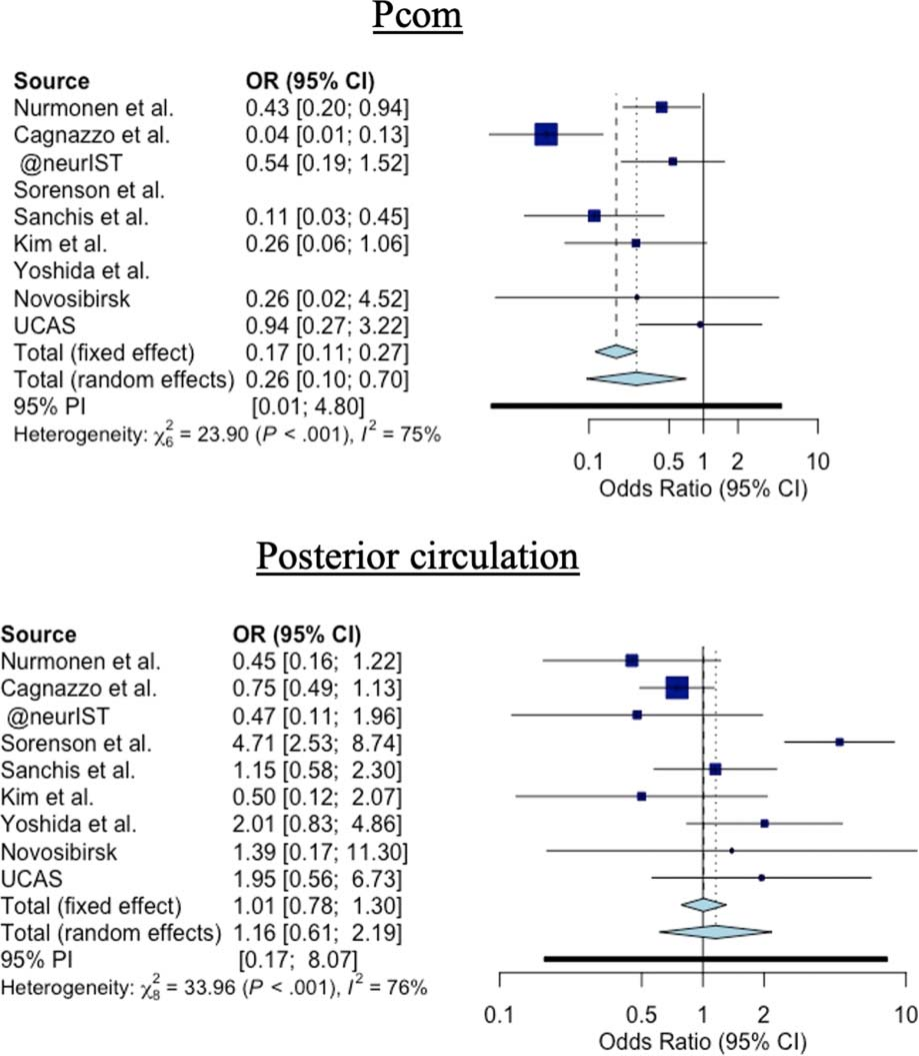
**Forest plot of the IA location distribution at the posterior circulation level.** A fixed effect model was chosen for an interstudies heterogeneity I^2^≤70% and a random effect model for a I^2^>70%. An odds ratio >1 favors the location to Autosomal dominant polycystic kidney disease (ADPDK) condition. CI, confidence interval; OR, odds ratio; Pcom, posterior communicating artery; UCAS, Unruptured Cerebral Aneurysms Study.

To investigate further the difference in IA locations between ADPKD and non-ADPKD patients, we have compared IA locations between these two groups for each study/cohort (Figures 3 and 4). The study conducted by Wilkinson *et al.* did not report IA location and has not been considered for this analysis.^31^ Regarding the anterior circulation locations (Figure 3), the meta-analysis revealed an OR in favor of patients with APDKD regarding the ICA location (OR: 1.90, 95% confidence interval [CI]: 1.10 to 3.29 in the random effect model). The same founding was observed for the MCA location (OR 1.18, 95% CI: 1.02 to 1.36, fixed effect model). No significant association was found the for Acom and Peri locations.

Concerning the Pcom and posterior locations (Figure 4), IAs in the non-ADPKD group were more associated with the Pcom location than IAs in the ADPKD group (OR: 0.31, 95% CI: 0.11 to 0.88, random effect model). Finally, the PCir location showed no significant result for ADPKD-IAs compared with non–ADPKD-IAs, with an OR 0.95 (95% CI, 0.74 to 1.22, fixed effect model).

## Discussion

We present here a meta-analysis comparing IAs location of patients affected or not by ADPKD. Data were extracted from seven articles published between 2016 and 2020 and combined to data from 3 additional cohorts: @neurIST (Switzerland), Novosibirsk (Russia), and UCAS (Japan). The ADPKD group contained more smokers, patients presenting hypertension, patients having multiple IAs, or having a positive family history for IAs than the non-ADPKD group. We showed that in patients affected by ADPKD, the frequency of IAs location in MCA, ICA, and Acom is higher in comparison with non-ADPKD patients. Thus, patients with ADPKD presented less IAs located on Pcom or Peri.

It has previously been described that IAs in patients with ADPKD patients are more frequently found within the anterior circulation.^32–34^ Our analysis confirms such observations and emphasizes that IAs in patients with ADPKD are more particularly found on large caliber arteries of the anterior circulation, mostly ICA and MCA, in comparison with non-ADPKD patients. Concerning the PCir, 14.3% of the IAs observed in non-ADPKD patients are located at the Pcom, which is in accordance with the UCAS^19^ and the International Study of Unruptured Intracranial Aneurysms cohorts.^20^ Interestingly, in our cohort of patients with ADPKD, only 2.6% of the IAs are diagnosed at this location. Finally, a larger proportion of IA was observed in the Acom, the last large caliber artery of the anterior cerebral trunk, for the ADPKD group, however, without reaching the level of significance.

The clinical relevance of these findings resides in the threshold after which a treatment should be proposed. Thus, the threshold at which non–ADPKD-IA located across large caliber arteries of the anterior circulation are considered at risk of rupture has been set at 7 mm.^28^ Because ADPKD-IAs present the tendency to rupture at smaller diameters^5,11,35^ and to show more IA multiplicity, the threshold should probably set at a lower size in patients with ADPKD.

IA development is influenced by several factors, an important one being WSS.^15–17^ Recent studies showed that IAs predominate in arteries with high velocity and at bifurcations where WSS is high.^36–39^ Arterial hypertension is a well-known major risk factor for IA development and rupture.^18,19,40^ Indeed, high WSS conditions favor leukocytes recruitment leading to internal elastic lamina disruption and formation of IAs. Because of the progressive renal dysfunction, patients with ADPKD present a higher rate of hypertension than the general population (Table 2). However, as Niemczyk *et al.*^40^ pointed out, it is very unlikely that arterial hypertension is the only and major factor explaining the higher frequency of IA development compared with the general population. The authors analyzed the blood pressure pattern between ADPKD patients with and without IA as well as the blood pressure variation during the day and the night. They concluded that arterial hypertension and high variation in blood pressure may influence IA development but should not be considered as a necessary factor for IA development. In patients with ADPKD, IAs are more frequently found in large caliber arteries with rare small bifurcations where WSS is low. In this sense, high blood pressure in patients with ADPKD represents more a marker of the severity of the systemic cardiovascular dysfunction than a potent inducer of IA development. One sensor of WSS in endothelial cells is primary cilia whom the expression and function are controlled in part by the polycystin-1 and polycystin-2 proteins. However, their exact roles for the physiology of the cerebrovascular tree and for pathologic disorders affecting cerebral arteries remain unclear. Extrapolating their mechanosensing role in kidney cilia, it can be postulated that primary cilia absence or dysfunction in patients with ADPKD favors wall fragility of the cerebral arteries.^14^ In a recent study, Diagbouga *et al.*^41^ conducted a histopathologic analysis in which they compared aneurysm domes located on MCA between ADPKD and non-ADPKD patients. They showed that in comparison with non–ADPKD-IA domes, ADPKD-IA domes were thinner, contained less collagen, and had a higher frequency of extremely thin thrombosis-lined hypocellular wall which are characteristics of severe IA wall deterioration.^41^

Because of the singular IAs distribution and characteristics of patients with ADPKD, the questions of which specific follow-up patients with ADPKD should receive and when their IAs should be treated have to be answered. The actual guidelines for IA screening in patients with ADPKD are controversial and differ between specialist societies.^1,11,31,42,43^ However, recent publications have highlighted a benefit of a presymptomatic screening in patients with ADPKD. Sanchis *et al.*, a nephrologic study group, reviewed 3010 patients with ADPKD from 1989 to 2017 of whom 812 patients without neurologic symptoms were screened with magnetic resonance angiography.^11^ They showed that 9.2% of the patients with ADPKD presented at least one IA and 1.7% had an IA ≤2 mm. The follow-up with magnetic resonance angiography allowed them to detect 1.07 *de novo* IAs per 100 patient-years and aneurysm growth in 13% of cases.^11^ The authors concluded that a presymptomatic screening was useful in their cohort. Similarly, Flahault *et al.* analyzed the cost-effectiveness of a presymptomatic screening in an ADPKD population.^44^ They concluded that a systematic presymptomatic screening was cost-effective and provided a gain of 0.68 quality-adjusted life years compared with targeted screening. The singular location distribution of IA among patients with ADPKD and their tendency to rupture at smaller diameter, we advocate for large IA screening and close imaging follow-up in all patients with ADPKD.

### Limitations

The main limitation of this study resides in the heterogeneity observed between studies (Figures 3 and 4) as assessed by a moderate to high I^2^ value.^29,45^ To overcome this limitation, a prospective observational study on patients with ADPKD with IA should be conducted. Assessing the IA rupture rate in patients with ADPKD in a followed up cohort might be highly biased by patient selection and ethically compromised.

Another limitation is the lack of information in the literature of the renal function at the time of IA diagnosis in patients with ADPKD, as well as the transplant status. A prospective study on patients with ADPKD harboring IAs should be conducted, with close monitoring of the renal function.

Finally, as patients with ADPKD represent a small proportion of the general population,^46^ our study compares a relative low number of patients with ADPKD harboring IA to a higher number of non-ADPKD patients.

In this study, we showed that IAs location distribution in patients with ADPKD differ from the ones in non-ADPKD patients. IAs in patients with ADPKD are more commonly located in the anterior circulation and in large caliber arteries. The ADPKD population more often presents with multiple IAs compared with the non-ADPKD individuals. Because of IA multiplicity and singular IA location distribution, patients with ADPKD represent a special neurovascular population who need to be closely followed.

## Supplementary Material

SUPPLEMENTARY MATERIAL

## Supplemental Material

Supplemental Table 1. International Stroke Genetic Consortium—Aneurysm Group.
